# Haploidentical stem cell transplantation in two children with mucopolysaccharidosis VI: clinical and biochemical outcome

**DOI:** 10.1186/1750-1172-8-134

**Published:** 2013-09-05

**Authors:** Sandra Jester, Julia Larsson, Erik A Eklund, Domniki Papadopoulou, Jan-Eric Månsson, Albert N Békássy, Dominik Turkiewicz, Jacek Toporski, Ingrid Øra

**Affiliations:** 1 Department of Pediatrics, Clinical Sciences, Lund University, Lund, Sweden; 2 Section for Experimental Pediatrics, Clinical Sciences, Lund University, Lund, Sweden; 3 Department of Pediatric Endocrinology, Skåne University Hospital, Lund, Sweden; 4 Department of Clinical Chemistry, Institute of Neuroscience and Physiology, Sahlgrenska Academy, University of Gothenburg, Gothenburg, Sweden; 5 Department of Pediatric Oncology and Hematology, Skåne University Hospital, Lund, Sweden; 6 Department of Oncogenomics, Academic Medical Center, University of Amsterdam, The Netherlands

**Keywords:** Mucopolysaccharidosis VI, Haploidentical stem cell transplantation, Clinical outcome

## Abstract

**Background:**

Mucopolysaccharidosis VI (MPS VI) is an autosomal recessive progressive multiorgan disorder due to mutation in the gene encoding the enzyme Arylsulfatase B (ARSB). Dysfunctional ARSB causes lysosomal accumulation of glycosaminoglycans (GAG). Currently, enzyme replacement therapy (ERT) is preferred to hematopoietic stem cell transplantation (SCT) due to the treatment-related risks of the latter. However, ERT constitutes an expensive life-long treatment. Increased experience and safety of SCT-procedures in recent years suggest that SCT should be further explored as a treatment option.

This is the first report on haploidentical SCT in patients with MPS VI. The primary objective was to assess the treatment safety and clinical and biochemical outcome.

**Patients and methods:**

Two siblings diagnosed with MPS VI at 10 months of age and at birth with genotype p.C192R, reported as mild to intermediate phenotype, underwent unrelated umbilical cord blood transplantation pre-symptomatic. Due to graft failure, both patients were urgently re-transplantated with haploidentical SCT with the father as donor. Continuous clinical and biochemical status was monitored and concluded 3.8 and 4.6 years after the haploidentical SCT.

**Results:**

Haploidentical SCT resulted in prompt and sustained engraftment. Complete donor chimerism was achieved in both patients, apart from mixed B cells chimerism in patient 2. ARSB activity in leukocytes post transplant increased from 0.0 to 19.0 μkat/kg protein (patient 1) and from 3.6 to 17.9 μkat/kg protein (patient 2) (ref. 17–40). Total urinary GAG normalized in both patients, although patient 2’s values slightly exceed normal range since 6 months. However, dermatan sulfaturia was substantially normalized since 16 months and 12 months post-SCT, respectively. Height was -1.85 SD and -1.27 SD at follow-up. Patient 1 had impaired visual acuity and discrete hepatomegaly. Patient 2 had elevated intraocular pressure and X-ray revealed steep acetabular angles and slightly flattened lumbar vertebrae.

**Conclusion:**

This study demonstrates that young children with MPS VI tolerate haploidentical SCT. Normalization of enzyme production and dermatan sulfaturia indicates correction of the inborn error of metabolism and coincide with no obvious symptoms of progressive MPS VI up to 4.6 years post-SCT.

## Background

Mucopolysaccharidosis type VI (MPS VI; Maroteaux-Lamy syndrome; MIM ID #253200) is an autosomal recessive lysosomal storage disorder, caused by mutations in the gene encoding arylsulfatase B (ARSB; N-acetylgalactosamine 4-sulfatase) [1]. ARSB is required for the degradation of the glycosaminoglycan (GAG) dermatan sulfate (DS), and the related GAG chondroitin sulfate [2]. ARSB-deficiency will ultimately lead to accumulation of these GAGs and their excretion in the urine [3,4].

The incidence of MPS VI varies greatly depending on ethnicity; from 1 in 38 563 live births among Turkish immigrants in Germany to 1 in 1 428 571 in Sweden [5,6].

### Clinical presentation and course

The great variety of clinical features usually includes short stature, hepatosplenomegaly, cardiac valve abnormalities, coarse facial features, joint contractures, hearing loss, upper airway obstruction, and claw-hand deformities [2,7-9]. Further skeletal abnormalities are hip and femoral head dysplasia and flattening of the vertebral bodies of the spine [10]. Visual impairments such as hypermetropia, high intraocular pressure, and glaucoma are common features [2,10], and essentially all MPS VI patients have some degree of corneal clouding [8,11]. In contrast to some other MPS disorders, the MPS VI patients are most often intellectually intact [2,7].

MPS VI patients can be sub-divided based on clinical features and age at onset of symptoms into mild, intermediate, or severe [12-15] and rapidly or slowly progressing [1-3]. The severe phenotype (rapidly progressing) manifests most often before 3 years of age, and the patients rarely grow taller than 120 cm [3]. If the disease remains untreated, death usually occurs by 2nd or 3rd decade of life, mainly due to heart failure or infections. Patients with slowly progressing MPS VI may remain undiagnosed until late teens or adulthood [2]. They have a longer life expectancy, often into the 5th or 6th decade of life [16] and reach near normal/normal height with mild skeletal and facial deformities. However, similar to the aggressive form they subsequently develop severe morbidity [3,13].

### Molecular genetics

Currently, 146 disease-causing ARSB mutations are identified in The Human Gene Mutation Database. The majorities are missense and nonsense mutations, the rest are deletions, insertions, splicing, and rearrangement mutations [2,4,15]. In general, truncating nonsense mutations and deletions together with missense mutations that directly affect the active site of the ARSB enzyme cause a more severe disease [4,15]. Due to the small number of MPS VI patients relative to the rather large number of mutations, genotype-phenotype predictions have been difficult to perform [4]. The homozygous missense mutation in the two patients described here, p.C192R, has been associated with a mild [12] or an intermediate severity [15], or a slowly progressing disease [13].

### Diagnostics of MPS VI

Early diagnosis and treatment in addition to progression rate are the most important prognostic factors [2,17,18]. Clinical suspicion of any type of MPS should immediately trigger qualitative and quantitative analyses of the uGAG, considering uGAG varies with age [19]. uGAG alone is not sensitive enough to dismiss the diagnosis if suspicion is high and, therefore, the current recommendations for diagnostic tests for MPS VI include enzyme activity of ARSB in leukocytes and/or fibroblasts and mutation analysis of the ARSB gene [2,17,19].

### Therapeutic alternatives

The current therapeutic options, in addition to symptomatic treatment, are enzyme replacement therapy (ERT) and hematopoietic stem cell transplantation (SCT) [2,10,18]. The potential benefits of both therapeutic options include improvements in clinical status and stabilization and/or delay of disease progression [18].

The primary aim of this paper was to report the clinical outcome of two siblings diagnosed with MPS VI who following unsuccessful umbilical cord blood transplantation (UCBT) were rescued with haploidentical stem cell transplantation (haplo-SCT). This is, to the best of our knowledge, the first report on haplo-SCT in MPS VI patients.

## Methods

Diagnosis, treatment, and follow-up was done at the Pediatric Oncology and Pediatric Endocrinology Units at Skåne University Hospital in Lund, Sweden. Examinations performed pre-transplant and at follow-up were: length/height, weight, and head circumference compared to WHO child growth standards, audiogram, ophthalmological examination (including Hooper Vision Organization Test, corneal examination and tonometry), echocardiography, standard 12-lead electrocardiogram, abdominal ultrasound, radiology (hand, hip and spine), articular examination, teeth-mouth status, as well as a detailed neurological examination. Photographic images of the patients were taken for documentation at assessment. Magnetic resonance imaging of the brain and spinal cord were performed before transplantation and revealed no signs of disease specific pathology. The standard 6-minute walk test (6MWT) could not be performed due to cooperative problems. However, the patients were observed during a 6-minute walk with their parents, a distance of 420 meters. Height of both parents was measured.

uGAG measurements and ARSB-activity were carried out at the Department of Laboratory Medicine/Clinical Chemistry, Sahlgrenska University Hospital, Gothenburg, Sweden. The mutation analysis was performed at Departement de Genética, Facultat de Biologia, Barcelona and at Skåne University Hospital, Lund, Sweden. Quantitative and qualitative uGAG-analyses were performed at the time of diagnosis, before the transplantations, one year post transplant, and then 2–4 times annually. The uGAG values were assessed in relation to the age-dependent normal reference ranges (ref.). ARSB activity in fibroblasts (ref. 40–90 μkat/kg protein) and leukocytes (ref. 17–40 μkat/kg protein) was measured at the time of diagnosis, thereafter in leukocytes 4 weeks after the haplo-SCT, 2–3 times annually for 3 years, and then annually.

Chimerism analysis was performed at regular intervals by amplification of variable number of tandem repeats as polymorphic genetic markers in selected cell populations (T- and B lymphocytes and myeloid cells). Immunological recovery was assessed by serology and lymphocyte distribution in peripheral blood.

### UCBT

After confirmed diagnosis of MPS VI, international expertise recommended SCT. The patients received unrelated UCBT, as no HLA-matched donor could be identified. Donor search and identification was carried out by the national Swedish Stem Cells Donor Registry at Karolinska University Hospital, Stockholm, Sweden. Both patients received myeloablative conditioning regimen consisting of busulfan without therapeutic drug monitoring (1.2 mg/kg iv every 6 hours for 16 doses on days -10 through -6), cyclophosphamide (50 mg/kg iv daily from day -5 through -2), and rabbit anti-thymocyteglobulin (thymoglobuline 2.5 mg/kg daily from day -3 through day -1). Ciclosporin and methylprednisolone were given as GvHD prophylaxis.

Details regarding the UCBT including cell doses are shown in Table 1.

**Table 1:** Details of the stem cell transplantations (SCT)

	**Pat1 (male)**	**Pat2 (female)**
**Age at diagnosis**	10 months	0 months
**Age at umbilical-SCT**	20 months	15 months
**Conditioning regimen umbilical-SCT**^**1**^	Bu/Cy/Thy	Bu/Cy/Thy
**HLA match**	6/6	4/6
**TNC (×10**^**6**^**/kg)**	75	44
**CD34+ (×10**^**6**^**/kg)**	1.2	0.26
**GvHD prophylaxis**	Ciclosporin and methylprednisolon	
**Outcome**	Graft rejection day +39	Non engraftment with autologous reconstitution day +42
**Time from umbilical- to haplo-SCT**	54 days	59 days
**Age at haplo-SCT**	22 months	17 months
**Cell source**	Peripheral blood stem cells from father	
**CD34+ (10**^**6**^**/kg)**	27	15
**CD3+ (10**^**6**^**/kg)**	0.047	0.0024
**T-cell depletion**	CliniMACS selection of CD34+ cells [20]	
**B-cell depletion**	Rituximab *in vivo* day +1	
**Conditioning regimen haplo-SCT**^**1**^	Flu/Eto/OKT3	Flu/TT/Mel/OKT3
**GvHD prophylaxis**	Mycophenolate mofetil day -1 to +28	
**Engraftment ANC > 500/ml**	Day +10	Day +16
**Complications**	None	Encephalitis of unknown origin - recovered

*TNC* total nucleated cells, *GvHD* graft versus host disease, *ANC* absolute neutrophil count, *Bu* busulfan, *Cy* cyclophosphamide, *Thy* thymoglobuline, *Flu* fludarabine, *Eto* etoposide, *OKT3* muromonab, *TT* thiotepa, *Mel* melphalan. ^1^ for doses, see Methods.

### Haplo-SCT

Both children experienced graft failure and, after consultation with EBMT (European Group for Blood and Marrow Transplantation), they were rescued with haplo-SCT 54 and 59 days after UCBT. Patient 1 (Pat1) underwent reconditioning with fludarabine (25 mg/m^2^ daily from day -4 through -1) and etoposide (10 mg/kg on day -1). Patient 2 (Pat2) was reconditioned with a more intensive regimen containing fludarabine (40 g/m^2^ daily from day -7 through -4), thiotepa (5 mg/kg on day -3), and melphalan (120 mg/m^2^ on day -2).

As rejection prophylaxis CD3 antibody muromonab was administered on days -8 to +15. Mycophenolate mofetil 600 mg/m^2^ bid was given on days -1 to +28 as GvHD prophylaxis. To reduce the risk of EBV-associated lymphoproliferative disease, *in vivo* B cell depletion was performed by a single infusion of 375 mg/m^2^ rituximab on day +1.

The father was chosen as an optimal donor. Donor peripheral blood stem cells were mobilized with recombinant human granulocyte-colony stimulating factor and harvested. The harvest was processed using immunomagnetic selection of CD34+ cells (CliniMACS system, Miltenyi Biotec, Germany) [20]. The dose of CD34+ for Pat1 was limited to 27×10^6^ /kg to reduce the risk of engraftment syndrome and the residual was stored in nitrogen liquid and transplanted to Pat2 9 months later. Details regarding the haplo-SCT are presented in Table 1.

## Patients

### Patient 1 (male)

Pregnancy and delivery were normal for Pat1. Birth weight was 3200 g (-0.31 SD), length 50 cm (+0.06 SD) and head circumference 33 cm (-1.15 SD). He was accidentally diagnosed with MPS VI at the age of 10 months, when hypergranulated neutrophils were detected on routine blood smear. uGAG was at two separate occasions 52.5 and 102 g/mol creatinine (ref. 22–50) with increased DS to 50% of total uGAG (ref. <10%). ARSB activity was 0 μkat/kg protein in leukocytes (ref. 17–40) and 11 μkat/kg protein in fibroblasts (ref. 40–90). Intellectual and physical development at the time of diagnosis was normal. Pat1 was treated for obstructive bronchitis in connection with upper airway infection at 10 months of age. The only pre-transplant findings consisted of a borderline hepatomegaly. Genetic analysis of the ARSB gene identified a homozygous p.C192R mutation.

### Patient 2 (female)

The younger sibling (Pat2) was born after a normal pregnancy and delivery. Her birth weight was 2860 g (-0.85 SD), length 47 cm (-1.15 SD) and head circumference 33 cm (-0.74 SD). Screening with uGAG immediately after birth revealed 227 g/mol creatinine (ref. 35–73). DS was increased to 50% of total uGAG (ref. <10%). ARSB activity in leukocytes was 3.6 μkat/kg protein (ref. 17–40). Pat2 harbored the homozygous mutation p.C192R, identical to that of Pat1. During the physical exam before the first transplantation, the only finding was irregular skeletal maturation on X-ray of the hands.

### Parents

The siblings were born to healthy parents of Arabic origin who were first cousins. Consanguinity was common in the family. Anamnestic information revealed six relatives with symptoms compatible with MPS VI (not confirmed by laboratory nor clinical examinations). The mother’s height was 162 cm (-0.18 SD) and the father’s 179 cm (+0.34 SD). The leukocyte ARSB activity was 25.4 μkat/kg protein for the father and 32.7 μkat/kg protein (ref. 17–40) for the mother. Both parents were confirmed heterozygous carriers of the mutation p.C192R. Informed consent for publication of the study was obtained from both parents.

## Results

### Haplo-SCT Pat1

After UCBT the patient suffered from multiple viral reactivations (EBV, Parvovirus B19, and HSV1) that induced hemophagocytic lymphohistiocytosis resulting in graft rejection 39 days post transplant. HSV1 pneumonitis and increasing respiratory obstruction necessitated mechanical ventilation. For life-saving reason, urgent haplo-SCT was performed with the father as donor, 54 days after the UCBT. The procedure was concluded without complications and donor-derived hematological recovery was achieved at day +10. The patient’s general condition improved quickly allowing discharge from the hospital at day +46 post haplo-SCT. Details of the transplantations are presented in Table 1.

### Haplo-SCT Pat2

Pat2 experienced primary non-engraftment with autologous recovery post-UCBT, confirmed by serial chimerism analysis. 59 days post-UCBT the patient received haplo-SCT with the father as donor, as no HLA-matched donor could be identified. Allogeneic recovery was achieved at day +16. Pat2 experienced one general seizure post haplo-SCT and subsequent MRI revealed a low attenuating area size 3×3 cm in the right frontal cerebrum. Lumbar puncture showed a minimal increase of lymphocytes, a few monocytes and atypical lymphoid cells. Cerebral biopsy was performed twice and showed inflammatory encephalopathy but no etiologic agent was identified. The recent MRI showed a decrease in the size of the area. No sequela has been observed to date. Details of the transplantations are shown in Table 1.

**Figure 1:**
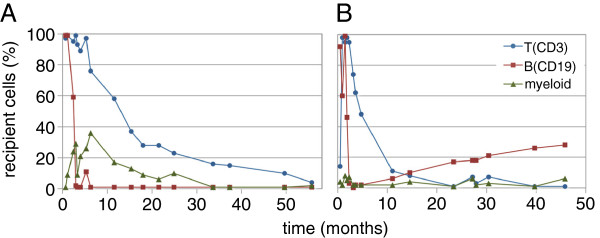
**Chimerism analysis of peripheral blood over time for both patients. A** = patient 1, **B** = patient 2. The percentage of recipient CD3+ T-cells (blue circles), CD19+ B-cells (red squares) and myeloid cells (green wedges) are shown from haploidentical stem cell transplantation to current assessment.

Initial analyses after haplo-SCT showed mixed chimerism in all cell lines in both patients, which subsequently converted to complete donor chimerism, except for B cells in Pat2 (Figure 1). Donor lymphocyte infusion was not required. Analyses of peripheral blood confirmed sustained immunological recovery for both patients.

### Urinary GAG

**Figure 2:**
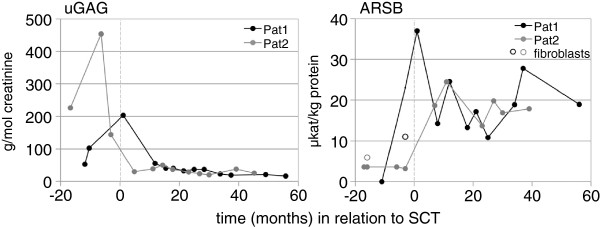
**Urinary glycosaminoglycan (uGAG) and leucocyte arylsulfatase B (ARSB) from MPS VI diagnosis to current assessment.** 0 = time of haploidentical SCT. Pat = patient. The non-filled circles of ARSB values are from fibroblasts. The amount of dermatan sulfate was normalized 16 and 12 months post haploidentical stem cell transplantation in Pat1 and Pat2, respectively.

Sixteen months post-haplo-SCT total uGAG in Pat1 was 39.7 g/mol creatinine (ref. 16–41) and DS was <10% which is within normal limits (Figure 2). Since then total uGAG has remained between 16–40 g/mol creatinine and contained normal amounts of DS. Pat2’s total uGAG had decreased to 38.3 g/mol creatinine (ref. 16–41) 12 months post-haplo-SCT and DS was <10%. Total uGAG has since then remained between 19–50 g/mol creatinine with normal DS.

### ARSB activity

The ARSB activity in leukocytes in Pat1 increased after the haplo-SCT and has been 10.8–37 μkat/kg protein with a mean of 20.5 (ref. 17–40) (Figure 2). In Pat2 the ARSB activity in leukocytes has been 13.7–24.5 μkat/kg protein with a mean of 18.6 (Figure 2).

### Current status of both patients

At follow-up assessment, Pat1 was aged 6.4 years and Pat2 was 5.2 years. They showed normal social skills and performed well in school and pre-school. Both patients received corticosteroid inhalation during upper airway infections to avoid wheezing. Both were on low dose levothyroxine substitution for mild hypothyroidism, diagnosed at 15 (Pat1) and at 7 (Pat2) months post-haplo-SCT. Neither hypothyroidism nor asthma or allergy was common in the family’s medical history. Pat1used glasses for astigmatism and hyperopia, and had esotropia of the left eye.

**Figure 3:**
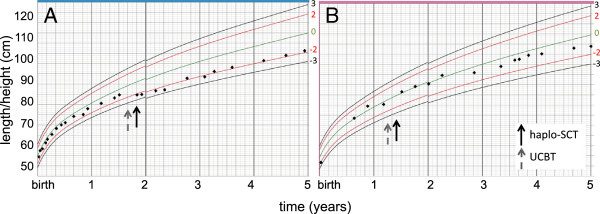
**Length/height development from birth to 5 years of age in both patients. A** = patient 1, **B** = patient 2. The black arrows indicate time for haploidentical stem cell transplantation (haplo-SCT) and the grey arrows time for umbilical cord blood transplantation (UCBT). Measurements are compared to WHO growth standards; mean (green), +/-2SD (red) and +/-3SD (black).

The clinical status is shown in Table 2 for Pat1 at 4.6 (4 years and 7 months) years and for Pat2 at 3.8 (3 years and 9 months) years post haplo-SCT. Both patients were shorter than the average child (-1.85 and -1.27 SD, respectively). The growth rate of Pat1 dropped 0.89 SD after the transplantations (Figure 3) but then remained steady at a height of approximately -2 SD. Pat2 has been growing at approximately -1 SD in height since 2.5 years of age (Figure 3).

**Table 2:** Clinical status at follow-up

	**Pat1**	**Pat2**
**Age at follow up**	6.4 years	5.2 years
**Time from haplo-SCT**	4.6 years	3.8 years
**Clinical examination**		
Neurological	Normal	Normal
Joint range of motion^**1**^	Normal; soft and flexible	Normal; soft and flexible
Facial appearance	Normal	Normal
Claw hands	None	None
Teeth-mouth-status	Normal, prognathism	Normal
**Measurements**^**2**^		
Height (SD)	109.0 cm (-1.85)	104.0 cm (-1.27)
Weight (SD)	19.7 kg (-0.67)	16.8 kg (-0.67)
Head circumference (SD)	50.0 cm (-0.49)	48.0 cm (-1.35)
**Mother’s height (SD)**	162.0 cm (-0.18)	
**Father’s height (SD)**	179.0 cm (+0.34)	
**Endurance**	Normal	Normal
**Ophthalmology**	Astigmatism, hyperopia, esotropia	Normal
Visual Acuity right/left eye	1.0/1.0 cc	0.8/0.8
Corneal clouding	None	None
Intraocular pressure	Normal	Elevated
**Otology**		
Examination	Normal	Bilateral otosalphingitis
Audiometry	Normal	Impaired air conduction
		Normal bone conduction
**Cardiology**		
Echocardiogram	Normal	Normal
Electrocardiogram	Normal	Normal
Blood pressure	110/60 mmHg	110/50 mmHg
**Imaging**		
Ultrasound abdomen	Discrete hepatomegaly	Normal
X-ray hand	Normal	Normal
X-ray spine	Biconvex L1-vertebrae	Slightly flattened lumbar vertebrae
X-ray hip/pelvis	Normal	Steep acetabular angles
**Obstructive episodes**^**3**^		
before/after haplo-SCT	1/6	0/1
**Laboratory assessment**		
ARSB (μkat/kg protein)	19 (17–40)	18 (17–40)
uGAG (g/mol creatinine)	16.0 (6–21)	25.2 (6–21)
Dermatan sulfate	Normal	Normal
**Chimerism analysis**		
(% recipient cells)		
T cells (CD3)	4 ± 4	<1
B cells (CD19)	<1	28 ± 2
Myeloid cells	2 ± 2	6 ± 0

*cc* cum correctore, *ARSB* arylsulfatase B, *uGAG* urinary glycosaminoglycan, *SCT* stem cell transplantations, *SD* standard deviation.

^**1**^SHOULDER: flexion, extension, abduction, adduction. ELBOW: flexion, extension, supination, pronation. WRIST: flexion, extension, abduction, adduction. HIP: flexion, extension, internal rotation, external rotation. KNEE: flexion, extension ^**2**^SD according to WHO child growth standards for boys and girls ^**3**^Documented hospital visits.

**Figure 4:**
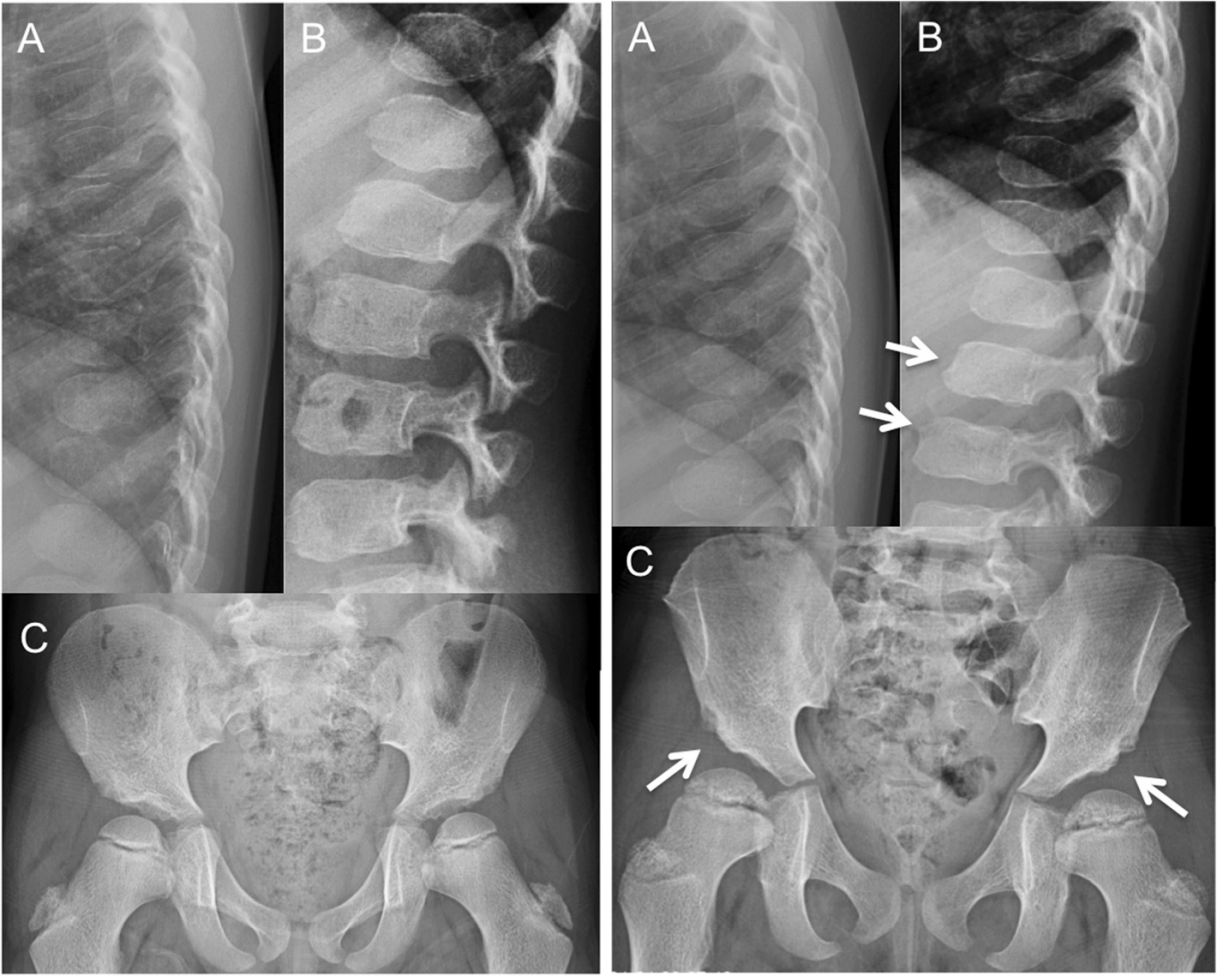
**X-ray of pelvis, thoracic and lumbar spine.** Left panel: Patient 1 (male, age 6.5 years) had a possible biconvex L1-vertebrae, although no obvious spinal pathology **(A, B)**. The hips are normal for age **(C)**. Right panel: Patient 2 (female, age 5.2 years) shows slightly flattened lumbar vertebrae, a possible pre-stage of anterior “beaking” typical for MPS VI (B, arrows), and bilateral steep acetabular angles (C, arrows).

Pat1 had remaining discrete hepatomegaly as seen before the treatment and X-ray on lumbar spine indicated a biconvex L1-vertebrae. Pat2 had now normal hands but slightly flattened lumbar vertebrae and bilateral steep acetabular angles (Figure 4) without associated symptoms. An elevated intraocular pressure and a bilateral otosalpingitis with a conductive hearing loss were found in Pat2 (Table 2).

**Figure 5:**
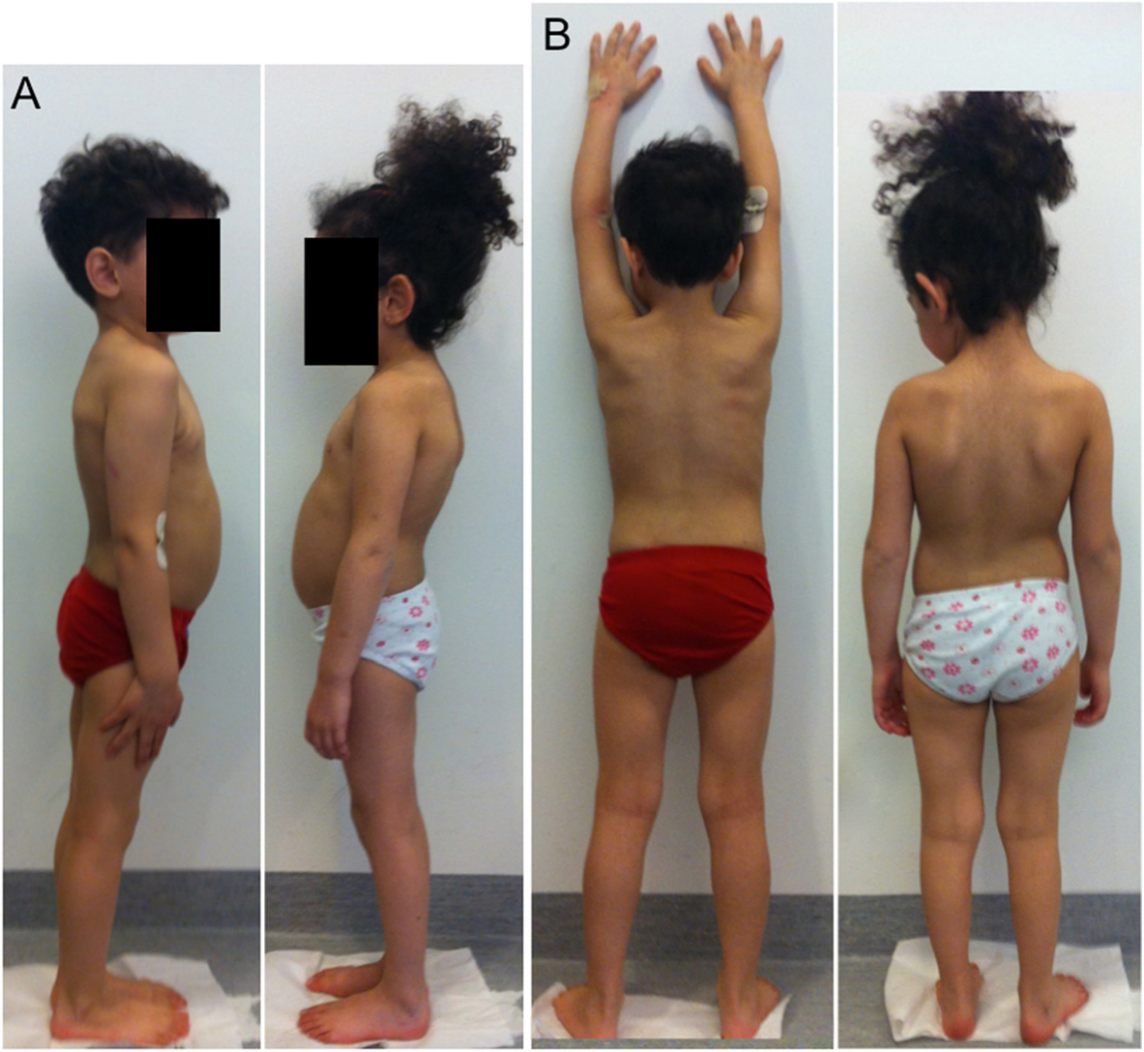
**Two patients with MPS VI, 4.5 and 3.75 years after haploidentical stem cell transplantation. Panel A:** lateral view, **Panel B:** posterior view; patient 1 to the left and patient 2 to the right. Physical appearance and motoric skills were normal for their age. The images are of low resolution for the patients’ confidentiality.

During the walk of 6 minutes (420 m), both patients had good endurance without any signs of fatigue, dyspnea, or difficulty with motor skills. Both were unaffected by running. Joint examination of the shoulder, elbow, wrist, hip and knee in the siblings revealed normal range of motion and no signs of pain or contractures. Photographs are seen in Figure 5.

## Discussion

Until 1984, MPS VI patients were considered palliative and only treated symptomatically [17]. Hematopoietic SCT for enzyme correction in MPS was introduced in 1982 [21]. More than forty-five patients with MPS VI have been reported transplanted so far [16]. Positive effects in MPS patients have been observed on enzyme activity and reduced uGAG [2], endurance, joint motility, puberty and growth, pulmonary/airway function, facial features, hepatosplenomegaly, and survival [16,17]. Hematopoietic SCT can, in contrast to ERT, improve ophthalmological and CNS abnormalities but the impact on cardiac disease remains unclear [9,17,21,22]. It cannot correct skeletal deformities that occurred prior to treatment, but may prevent further progression [16,17]. In the largest retrospective study of transplanted MPS VI patients to date an overall survival rate of 66% at 3 years post-transplant was found [16]. However the majority (64%) of those patients were treated before 2000 and the main cause of death was infection or organ failure. Given the continuous progress in supportive care, better donor selection, and new conditioning approach (e.g. busulfan monitoring), a significantly reduced procedure-related morbidity and mortality in connection with hematopoeical SCT would be expected today [23].

Transplantation from a haploidentical family donor has become an established procedure for treatment of children with otherwise incurable malignant diseases and serves as a treatment option for inborn or acquired immunodeficiencies and other genetic disorders [24]. In-vitro manipulation of the graft, mostly paternal/maternal, and improved supportive care has lead to improved outcome after haplo-SCT with decreased treatment-related toxicity and infections, compared to conventional SCT [25,26]. Haplo-SCT is in addition growing as an alternative to HLA-matched hematopoietic SCT in urgent cases.

ERT was developed in the 1990s and the treatment of MPS VI was introduced in 2005 [17]. Recombinant human ARSB are administered intravenously weekly or every second week. Long-term improvements regarding pulmonary function and growth rate have been observed [27,28]. However, the enzyme cannot reach the CNS, cornea, and articular cartilage [2]. The largest study of ERT-treated MPS VI-patients showed improvements concerning organomegaly, endurance and pulmonary function but no improvements in vision, hearing, or cardiac function [9]. It is important to consider the costs of ERT (€150, 000–450,000 annually per patient [29]) as well as the psychological burden of a life-long treatment. The efficacy of ERT in MPS VI still needs to be long term evaluated, as the treatment is only available since 2005.

The risks of SCT previously appeared to exceed the benefits compared to ERT, and has for some years been considered a second choice [10]. As a consequence, only few publications regarding treatment outcome have focused on hematopoietic SCT in MPS VI after introduction of ERT. As highlighted above, SCT has some clinical advantages compared to ERT and imposes a single cost as well as a time-limited therapy for the patient. For these reasons, it is of great importance to continue evaluation of advantages and disadvantages of these two treatment modalities.

The current patients are expected to develop disease specific symptoms later in childhood or adolescence considering the predicted mild to intermediate phenotype. One patient described in 2011 with homozygote mutation p.C192R had at the age of 19 developed severe skeletal and ophtalmological manifestations in addition to pulmonary, nervous and cardiac symptoms [13]. Here, the patients with the same genotype were doing well at assessment 5– and 6 years of age, which might reflect their phenotype of slowly progressing disease. Nevertheless, the biochemical analyses indicated sufficient ARSB enzyme production from the heterozygote p.C192R donor graft. They develop normally with good endurance and motility. The endurance test in this study was a simplified version of the standard 6MWT, nevertheless the patients walked a distance of 420 meters unaffected, which is considered normal/close to normal in 5 and 6 year-olds [30]. Pat1 had a relatively short stature. Although it may represent a symptom of MPS VI, more probably it is a consequence of treatment procedure, considering the halted growth at time of transplantation. X-ray on lumbar spine in Pat1 indicated a biconvex L1-vertebrae with possible association to MPS VI. Relevant is also Pat1’s impaired visual acuity (hyperopia and esotropia) and the discrete hepatomegaly. Most notable in Pat2 were the radiological, ophtalmological, and audiological findings, although the latter probably constitute a normal variant due to a recent otitis media. Lumbar X-ray on Pat2 showed anterior-inferior beaking typical for MPS VI [2,10]. The bilateral dysplastic hips are typical of MPS VI, but may also be an undiagnosed congenital defect or late skeletal development.

Both patients were at time of assessment treated for mild hypothyroidism and transient but recurrent wheezing. It is hard to truly evaluate whether these symptoms are related progression of MPS VI or long-term complications of treatment. It is therefore essential to report the current patients in a longer perspective.

In conclusion, two young children with MPS VI received and tolerated haploidentical SCT after previous transplant failure. Normalization of enzyme production and dermatan sulfaturia indicate correction of the inborn error of metabolism and coincide with no obvious symptoms of MPS VI progression up to 4.6 years post-SCT. The subtle clinical findings reported here need longer follow-up to determine their etiology. Additional data on clinical and biochemical outcome after SCT in MPS VI-patients is needed to motivate its role as adequate treatment and whether haploidentical SCT might be a future recommendation.

## Consent

Written informed consent was obtained from the father and the mother for publication of this report and accompanying images. A copy of the written consent is available by the Editor-in-Chief of this journal.

## Abbreviations

ARSB: Arylsulfatase B; DS: Dermatan sulfate; EBMT: European group for blood and marrow transplantation; ERT: Enzyme replacement therapy; GAG: Glycosaminoglycan; GvHD: Graft versus host disease; haplo-SCT: Haploidentical stem cell transplantation; MPS: Mucopolysaccharidosis; UCBT: Umbilical cord blood transplantation; uGAG: Urinary glycosaminoglycan; SD: Standard deviation; ref.: Normal reference range; 6MWT: Six minute walk test.

## Competing interests

The authors declare that they have no competing interests.

## Authors’ contributions

SJ, JL, JT, IØ initiated and designed the study, collected clinical data, and drafted the manuscript. All authors made substantial contributions to data analysis, manuscript edition and critical revision for important intellectual content. All authors read and approved the final manuscript.
